# Consideration of Viral Resistance for Optimization of Direct Antiviral Therapy of Hepatitis C Virus Genotype 1-Infected Patients

**DOI:** 10.1371/journal.pone.0134395

**Published:** 2015-08-28

**Authors:** Julia Dietz, Simone Susser, Caterina Berkowski, Dany Perner, Stefan Zeuzem, Christoph Sarrazin

**Affiliations:** Medical Department 1, Goethe University Hospital, Frankfurt, Germany; Kaohsiung Medical University Hospital, Kaohsiung Medical University, TAIWAN

## Abstract

Different highly effective interferon-free treatment options for chronic hepatitis C virus (HCV) infection are currently available. Pre-existence of resistance associated variants (RAVs) to direct antiviral agents (DAAs) reduces sustained virologic response (SVR) rates by 3–53% in hepatitis C virus (HCV) genotype 1 infected patients depending on different predictors and the DAA regimen used. Frequencies of single and combined resistance to NS3, NS5A and NS5B inhibitors and consequences for the applicability of different treatment regimens are unknown. Parallel population based sequencing of HCV *NS3*, *NS5A* and *NS5B* genes in 312 treatment-naïve Caucasian HCV genotype 1 infected patients showed the presence of major resistant variants in 20.5% (*NS3*), 11.9% (*NS5A*), and 22.1% (*NS5B*) with important differences for HCV subtypes. In *NS3*, Q80K was observed in 34.7% and 2.1% of subtype 1a and 1b patients, respectively while other RAVs to second generation protease inhibitors were detected rarely (1.4%). Within *NS5A* RAVs were observed in 7.1% of subtype 1a and 17.6% in subtype 1b infected patients. RAVs to non-nucleoside NS5B inhibitors were observed in 3.5% and 44.4% of subtype 1a and 1b patients, respectively. Considering all three DAA targets all subtype 1a and 98.6% of subtype 1b infected patients were wildtype for at least one interferon free DAA regimen currently available. In conclusion, baseline resistance testing allows the selection of at least one RAVs-free treatment option for nearly all patients enabling a potentially cost- and efficacy-optimized treatment of chronic hepatitis C.

## Introduction

Detailed characterization of viral proteins with critical functions in the hepatitis C virus (HCV) replication cycle, like the NS3/4A protease, the NS5A protein and the NS5B polymerase together with the invention of a cell culture replication model led to the development of direct acting antivirals (DAA) targeting these HCV proteins [1, 2]. Currently, different interferon-free combination therapies for treatment of chronic hepatitis C virus (HCV) infection with direct antiviral agents (DAAs) are approved. For HCV genotype 1 infected patients combination therapies of a nucleotide NS5B polymerase inhibitor (Sofosbuvir, SOF) with either a NS3 protease inhibitor (Simeprevir, SMV) or a NS5A inhibitor (Daclatasvir, DCV and Ledipasvir, LDV) are available [3–7]. Alternative options are a combination of a NS3 protease and NS5A inhibitor with restriction to HCV subtype 1b (Asunaprevir, ASV plus Daclatasvir) or a triple DAA therapy (NS3 protease-, NS5A- and non-nucleoside NS5B inhibitor) for all genotype 1 infected patients (Paritaprevir, PTV, Ombitasvir, OMV and Dasabuvir, DSV) [8–12]. Overall, high rates of sustained virologic response (SVR) between 82% and 99% have been observed in the different underlying studies [3–12]. Predictors of SVR mainly are non-response to previous antiviral therapy and the presence of liver cirrhosis [8, 9, 11, 12]. However, also pre-existence of resistance associated variants (RAVs) was associated with a reduction of SVR rates by 3–53% in studies with available data [5–8]. While the NS5B nucleotide analogue Sofosbuvir has a high genetic barrier to resistance and no clinical relevance of pre-existing L159, S282 and V321 variants for IFN-free therapies have been shown so far, for NS3 protease-, NS5A- and non-nucleoside NS5B-inhibitors, RAVs with different levels of resistance to the different available DAAs have been described and found clinically relevant [13–23].

In the present study, frequencies of RAVs to currently available NS3, NS5A and NS5B inhibitors have been assessed in 312 Caucasian patients with HCV genotype 1 infection by parallel population-based sequencing for the exploration of the rate of patients with coexistence of RAVs for different dual and triple DAA combination therapies currently available.

## Materials and Methods

### Patients

Baseline serum samples of 312 consecutive Caucasian patients, with a chronic genotype 1 hepatitis C infection who were treatment- and DAA-naïve were obtained from previously conducted clinical studies [24]. Investigations were performed according to the Declaration of Helsinki and approval of the enrollment in the respective studies as well as the usage of patient blood samples for research purpose was obtained from the local ethics committee (Ethikkommission der Ärztekammer des Saarlandes), and written informed consent was obtained from all patients.

### HCV RNA extraction, reverse transcription and PCR

HCV RNA was extracted from 140 μL serum (QIAamp Viral RNA Mini-Kit, Qiagen, Hilden, Germany) and complementary DNA (cDNA) was synthesized using SuperScript III Reverse Transcriptase (Invitrogen) as previously described [25]. For all amplifications of the respective HCV regions, we conducted nested PCRs using 1/10 of cDNA or outer PCR product respectively by applying the Fast Cycling PCR Kit (Qiagen). All PCRs were carried out by using the primers for both subtypes (1a and 1b) in combination. The HCV *NS3* protease domain was amplified in the outer PCR with primers described previously [26]: U376_1bc_F, ATGGAGACCAAGATCATCACCTGGG;U3276_1a_F, ATGGAGACCAAGCTCATCACGTGGG; D4421_1b_R, CCGTCGGCAAGGAACTTGCCATAGGTGGA and D4421_1a_R, ACCCGCCGTCGGCAAGGAACTTGCCGTA. First, PCR mixtures were incubated at 95°C for 5 minutes, followed by 35 cycles at 96°C for 5 seconds, 60°C for 5 seconds and 68°C for 1 minute and a final elongation occurred at 72°C for 1 minute. The inner PCR was performed with 3420_1b_F, AGGGCATTTAAATAGCCACCATGGCGCCCATCACGGCCTACTCCCAACAGAC; 3420_1a_F, AGGGCATTTAAATAGCCACCATGGCGCCCATCACGGCGTACGCCCAGCAGAC; 4038_1b_R, AAAAAGCGGCCGCAGCCGGCACCTTAGTGCTCTTGCCGCTGCC; 4038_1a_R, AAAAAGCGGCCGCAGCCGGGACCTTGGTGCTCTTACCGCTGCC and the same cycling conditions as for the outer PCR. *NS5A* was amplified using NS5A_1a_6279_F, ATCTGGGACTGGATATGC; NS5A_1b_6279_F, GTTTGGGACTGGATATGC; NS5A_1a_6599_R, AGACACCCTCCACAG and NS5A_1b_6650_R, CGTCACGTAGTGGAAATC in the outer PCR. The cycling conditions for *NS5A* were the same as for the amplification of *NS3*, but an annealing temperature of 50°C as well as a 30 second elongation step at 68°C were used. For the inner PCR, NS5A_1a_6285_F, GACTGGATATGCGAGGTG; NS5A_1b_6318_F, ACCTGGCTCCAGTCCAAG; NS5A_1a_6590_R, CCACAGCGCGAACKTATAG; and NS5A_1b_6618_R, CCTCCACRTACTCCTCAG were used. Besides the annealing temperature of 54°C, the PCR profile was the same as for the outer *NS5A* PCR. For the semi-nested amplification of *NS5B*, in the outer PCR, NS5B_1a_8445_F, AGCGGCGTACTGACAAC; NS5B_1b_8460_F, ACTAGCTGCGGCAACACC; NS5B_1a_1702_R, GGGCATGAGACACGCTGTG and NS5B_1b_1700_R, GCACGAGACAGGCTGTG primers were used. The inner PCR amplification was performed with both reverse primers of the outer PCR in combination with NS5B_1a_8467_F, GTGGTAACACCCTCACTTG and NS5B_1b_8522_F, GCTCCAGGACTGCACAATG forward primers. The temperature profile of both outer and inner *NS5B* PCRs was the same as for the amplification of *NS3*, but an annealing temperature of 52°C was applied for the inner and outer *NS5B* PCRs. The resulting PCR products were analyzed for correct size on 1% agarose gels stained with ethidium bromide and were gel-purified using the QIAquick Gel Extraction Kit (Qiagen).

### Sequencing analysis of HCV *NS3*, *NS5A* and *NS5B* genes

The purified PCR products of *NS3*, *NS5A* and *NS5B* were population-based sequenced. *NS3* of GT1a and 1b was sequenced with the inner forward PCR primer and *NS5A* sequencing occurred for GT1a with the inner forward and for GT1b with the inner reverse PCR primer. *NS5B* was sequenced using one forward primer for both genotypes (NS5B_1a/1b_1213F, GTCAATTCCTGGCTAGGC) and specific reverse primers for GT1a and GT1b which were also used for the amplification of *NS5B*. All sequencing analyses were performed according to the manufacturer´s protocol (Big Dye Terminator v1.1 Cycle Sequencing Kit, Applied Biosystems) on an ABI Prism 3130*xl* Genetic Analyzer (Applied Biosystems). Population-based sequencing generates a consensus sequence of the viral quasispecies and has a sensitivity of approximately 20% for minority variants. In accordance with another study [27] all RAVs observed at the respective positions in the electropherogram were taken into account. This included also minority variants which were recognized as mixed peaks in the sequence. All cases with minority variants are listed in the supplementary information (S1 Table). After proofreading, all sequences were aligned using BioEdit.version 7.2.3 [28].

### Analysis of baseline RAVs

We investigated the occurrence of baseline RAVs within samples of 312 HCV GT1-infected DAA-naïve patients for which parallel sequences for *NS3*, *NS5A* and *NS5B* were obtained (n = 170 GT1a, n = 142 GT1b). RAVs for currently approved for IFN-free treatment of chronic hepatitis C (SMV, ASV; PTV, DCV, LDV, OMV, DSV) were considered as relevant, if they were described in previous studies to be associated *in vivo* with treatment failure and / or have been shown in *in vitro* phenotypic assays to confer a more than 2-fold changed drug susceptibility in comparison to the wildtype reference strain. Based on this, RAVs were analysed at the following positions in comparison with the respective HCV reference strain (GT1a: H77 [29], GT1b: Con1 [30]): *NS3*: F43/I/L/S//V, Y56H, Q80K/R, S122R, R155K/G, A156G/S/T and D168A/C/E/G/H/N/T/V/Y; *NS5A*: L/M28A/T/V, Q30E/H/R and R30Q (secondary site), L31F/I/M/V, P32L, Q54H (secondary site), H58D and P58S (secondary site), Y93C/F/H/N/S; *NS5B*: C316H/N/Y, S368T, Y448C/H, S556G/R, D559R [17–22, 31–36]. Table 1 shows the EC50 values of variants which were detected in our cohort. Sequencing of variants associated with resistance to sofosbuvir *in vitro* and *in vivo* (L159F, S282T, V321A) was not performed as these variants were not detected before treatment initiation (S282T) [15, 16] and / or (L159F, V321A) were not associated with reduced susceptibility to sofosbuvir [16, 22, 37, 38]. All HCV subtypes were determined by sequencing analysis of the NS3 protease domain. We refer an HCV region as wildtype when the respective region does not contain RAVs according to our definition. We classified identified RAVs on the basis of fold change compared to the wildtype replicon determined in previous studies in low-level resistant (2-10-fold change), intermediate resistant (11-100-fold change) and high-level resistant (>100-fold change) variants (Table 1).

**Table 1:** EC50 values of baseline RAVs within *NS3*, *NS5A* and *NS5B* detected in the present cohort.

Position	Variant	HCV region	EC50 [fold-change]^1^ (subtype)	Resistance Level	DAA	References
Q80	K	*NS3*	9.3 (1a), 7.7 (1b)	low	SMV	[31]
			3 (1a), 6.5 (1b)		ASV	[32]
			3 (1a)		PTV	[34]
	R	*NS3*	13 (1a), 6.9 (1b)	low-intermediate	SMV	[31]
			4 (1b)		ASV	[32]
			2 (1a)		PTV	[34]
D168	E	*NS3*	26 (1a), 43 (1b)	low-intermediate	SMV	[31]
			58 (1a), 78 (1b)		ASV	[32]
			14 (1a), 4 (1b)		PTV	[34]
M28	V	*NS5A*	1.3 (1a)	intermediate	DCV	[19]
			n.d.		LDV	n.d.
			58 (1a)		OMV	[18]
Q30	H	*NS5A*	1477 (1a)	low-high	DCV	[19]
			73 (1a)		LDV	[21]
			3 (1a)		OMV	[36]
L31	M	*NS5A*	341 (1a), 3 (1b)	low-high	DCV	[19]
			140 (1a), 2.5–100 (1b)		LDV	[21, 22]
			2 (1a), 0.9 (1b)		OMV	[33]
	F	*NS5A*	5 (1b)	low	DCV	[20]
			n.d.		LDV	n.d.
			10 (1b)		OMV	[18]
Y93	C	*NS5A*	1864 (1a)	high	DCV	[19]
			327 (1a)		LDV	[21]
			1675 (1a)		OMV	[18]
	F	*NS5A*	n.d.	low-intermediate	DCV	n.d.
			2.5–100 (1a)		LDV	[22]
			n.d.		OMV	n.d.
	H	*NS5A*	5432 (1a), 24 (1b)	intermediate-high	DCV	[19]
			3309 (1a), 1319 (1b)		LDV	[21]
			41383 (1a), 77 (1b)		OMV	[36]
	N	*NS5A*	47477 (1a)	high	DCV	[19]
			>100 (1a)		LDV	[22]
			66739-fold (1a)		OMV	[18]
C316	H	*NS5B*	229 (1b)	high	DSV	[33]
	N	*NS5B*	5 (1b)	low	DSV	[35]
	Y	*NS5B*	1472 (1a), 1569 (1b)	high	DSV	[35]
Y448	H	*NS5B*	975 (1a), 46 (1b)	intermediate-high	DSV	[35]
S556	G	*NS5B*	30 (1a), 11 (1b)	intermediate	DSV	[35]
	N	*NS5B*	29 (1a)	intermediate	DSV	[35]
	R	*NS5B*	261 (1a)	high	DSV	[33]
C316N+S556G		*NS5B*	38 (1b)	intermediate	DSV	[33]

^1^ compared to WT replicon

n.d.: not determined

## Results

### Patient characteristics of the cohort

The baseline clinical characteristics of the investigated patients are shown in Table 2. The majority of patients were male (n = 207, 66.4%) and the median age was 47 years. The median HCV viral load was 1.4x10^6^ IU/mL and approximately half of the patients were infected with HCV subtype 1a and 1b, respectively. Information on *IL28B* genotype information was available for 273 individuals and a CC genotype was found in 94 patients (34.4%) and a liver cirrhosis was diagnosed in 17.6% (n = 48) of patients.

**Table 2:** Patient characteristics at baseline.

Parameter	Patients
Age (years) [median (range)]	47 (19–79)
Sex	
Male [n (%)]	207 (66.4%)
Female [n (%)]	105 (33.6%)
Body mass index (kg/m^2^) [median (range)]	24.5 (16.6–45.5)
*IL28B* CC genotype	
CC [n (%)]	94 (34.4%)
non-CC [n (%)]	179 (65.6%)
n.d. [n]	39
Liver enyzmes	
ALT (U/l) [median (range)]	78 (14–551)
AST (U/l) [median (range)]	56 (5–398)
GGT [median (range)]	63 (2–720)
Cirrhosis	
Patients with cirrhosis [n (%)]	48 (17.6%)
Patients without cirrhosis [n (%)]	225 (82.4%)
n.d. [n]	39
HCV infection	
HCV viral load (IU/mL) [median (range)]	1.4x10^6^ (5.2x10^3^–3.6x10^7^)
HCV GT 1a [n (%)]	170 (54.5%)
HCV GT 1b [n (%)]	142 (45.5%)

### Baseline *NS3* protease RAVs

**Fig 1:**
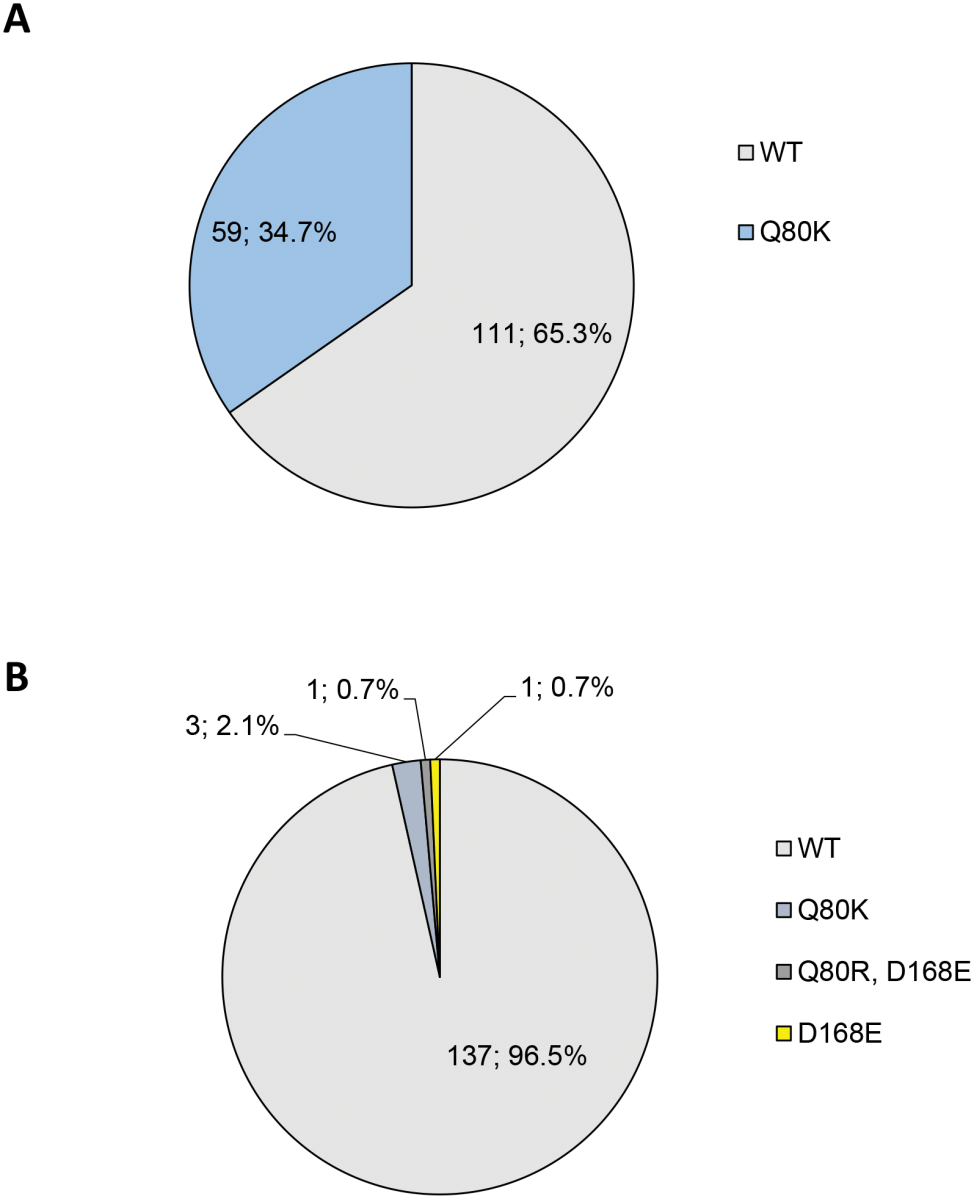
Baseline *NS3* RAVs in DAA-naïve patients. A) GT1a- and B) GT1b-infected patients.

The HCV *NS3* protease sequences were analyzed at positions which have previously been reported to be responsible for the emergence of RAVs during treatment with protease inhibitors (PIs). For the macrocyclic PIs simeprevir, asunaprevir and paritaprevir, which are approved for an interferon-free treatment of chronic hepatitis C, these primary resistance mutations were detected at positions Q80, R155 and D168 [39]. At baseline, we were not able to detect the intermediate resistance conferring R155K mutation and also R155G was not observed. Only two GT1b-infected patients (1.4%) displayed a D168E variant which is resistant to SMV, ASV and PTV (SMV: 43-fold, ASV: 78-fold, PTV: 4-fold change in EC_50_, Table 1). The Q80K variant conferring low-level resistance to SMV (in GT 1a and 1b) and ASV (in GT 1b) occurred at baseline in 19.9% (n = 62/312) of GT1-infected patients and was detected in 34.7% (n = 59) of GT1a- and in 2.1% (n = 3) of GT1b-infected individuals (Fig 1). However, Q80K confers only minimal resistance to PTV [34]. At position 80, only one individual with a GT1b infection had a Q80R variant (0.7%). The overall occurrence of *NS3* RAVs in GT1 was 20.5%.

Several other positions with varying relevance for different PIs have been reported and variants at positions F43 and A156 confer resistance *in vitro* to SMV and ASV but were not observed *in vivo* at baseline or in association with treatment failure [17, 27, 31, 32, 40, 41]. These variants, the SMV intermediate-resistant S122R variant as well as the Y56H mutation which occurred in combination with D168A in PTV-treated patients [11] and is known to be relevant for treatment with MK-5172 [27] were not detectable in the present cohort by baseline population-based sequencing.

### Baseline *NS5A* RAVs

Resistance mutations within *NS5A* which were described to confer resistance to daclatasvir, ledipasvir and ombitasvir are largely overlapping. These include the primary variants at positions M28, Q30, L31, and Y93 [42]. Secondary site mutations were described for daclatasvir with R30Q and P58S enhancing the resistance of primary mutations but which are not themselves resistant and Q54H was also reported as secondary mutation, not increasing the resistance of primary resistance mutations [20]. However, M28A/T, L31V and H58D variants were not detected in our cohort. For GT1a, these variants were shown to confer resistance towards DCV, LDV and OMV in studies with available data [18–20, 22, 36].

**Fig 2:**
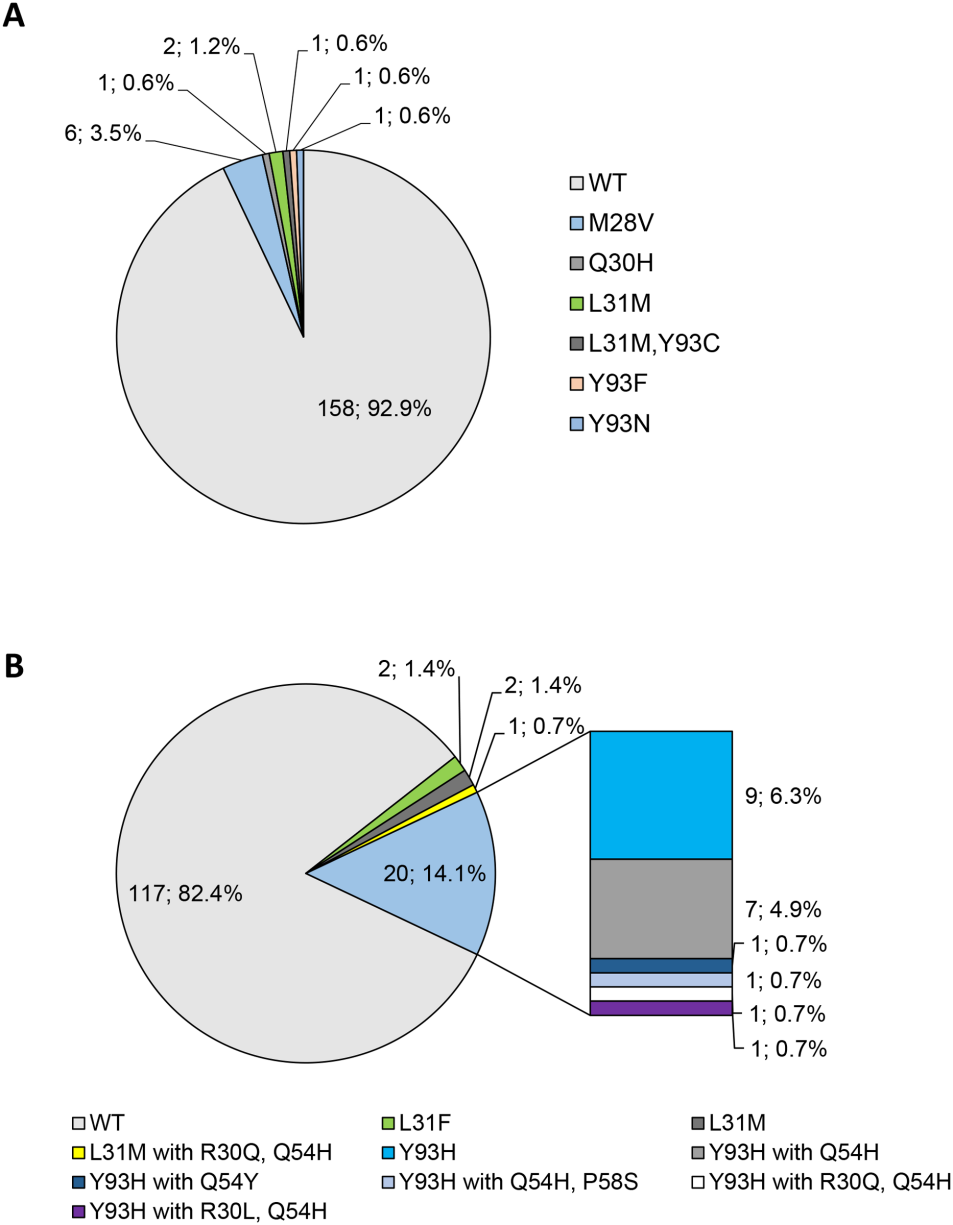
Occurrence of RAVs within *NS5A* in GT1a- (A) and GT1b- (B) infected patients.

In GT1a-infected patients, NS5A inhibitor resistant variants were detected rarely (7.1%) with the M28V variant conferring intermediate resistance towards OMV (not to DCV and LDV) occurring most frequently (3.5%) followed by DCV and LDV high level resistant L31M (1.2%) mutation. Further detected variants were Y93F/N, Q30H and L31M, Y93H (each 0.6%) (Fig 2A). In GT1b-infected patients, the prevalence of RAVs (17.6%) was higher in comparison to GT1a and the intermediate to high level Y93H variant was the primary mutation which emerged (Y93H: 14.1%), which was frequently accompanied by DCV secondary mutations at positions R30, P58 and Q54 (Fig 2B). In GT1 the total number of NS5A RAVs was 11.9%. Besides this, L31F/M variants were rarely found in GT1b (L31F: 1.4%, L31M and or with R30Q, Q54H: 2.1%). L31M confers no resistance towards OMV [36] and the resistance of L31F towards LDV is unknown so far.

### Baseline non-nucleoside inhibitor *NS5B* RAVs

**Fig 3:**
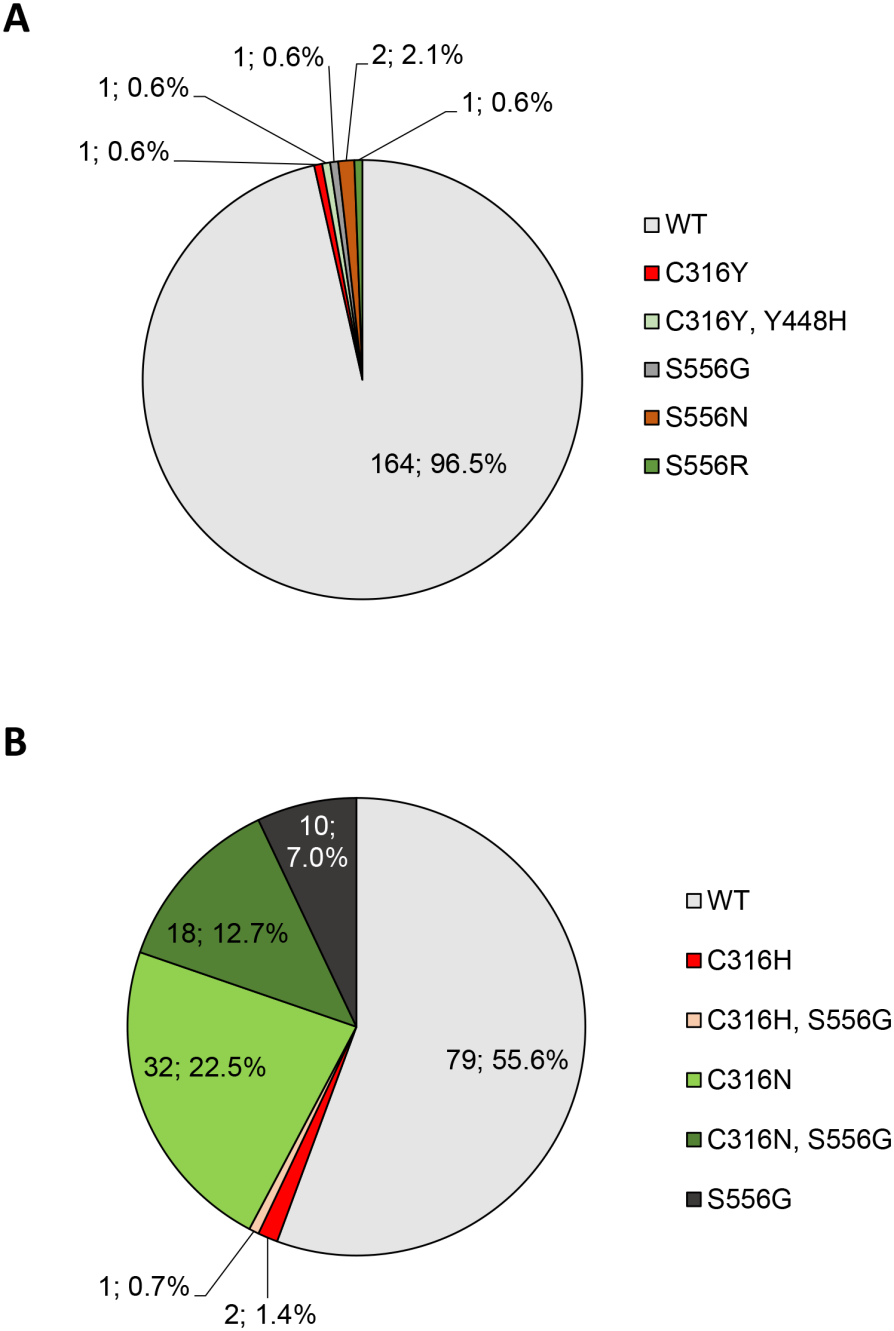
Prevalence of *NS5B* RAVs in GT1a- (A) and GT1b- (B) infected patients.

Within *NS5B*, we analyzed resistant variants known to be associated with dasabuvir treatment failure: C316H/N/Y, Y448H/C, S556G/N/R and D559G [11, 33, 35, 43]. The S368T mutation is high level resistant *in vitro* in GT1b against DSV [35] but was not found *in vivo* so far and was also not detectable in the present study. We observed *NS5B* RAVs in 22.1% of GT1 patients. Interestingly, the prevalence of DSV-resistant RAVs was low in GT1a-infected individuals (3.5%). Two patients each carried high-level resistant C316Y or intermediate resistant S556N (1.2%) and one individual each had intermediate resistant S556G or high level resistant S556R mutation (0.6%). By contrast, RAVs appeared frequently in 44.4% of GT1b infected patients and the dominating variants were C316N (22.5%), S556G (7.0%) as well as C316N+S556G (12.7%) (Fig 3). However, these mutations have been described to confer a relatively low resistance with a 5-fold (C316N) and 11-fold (S556G) resistance, respectively compared to a GT1b wildtype replicon. A combination of both mutations yielded in a 38-fold resistance [33, 35].

### RAVs with respect to different treatment regimens

**Fig 4:**
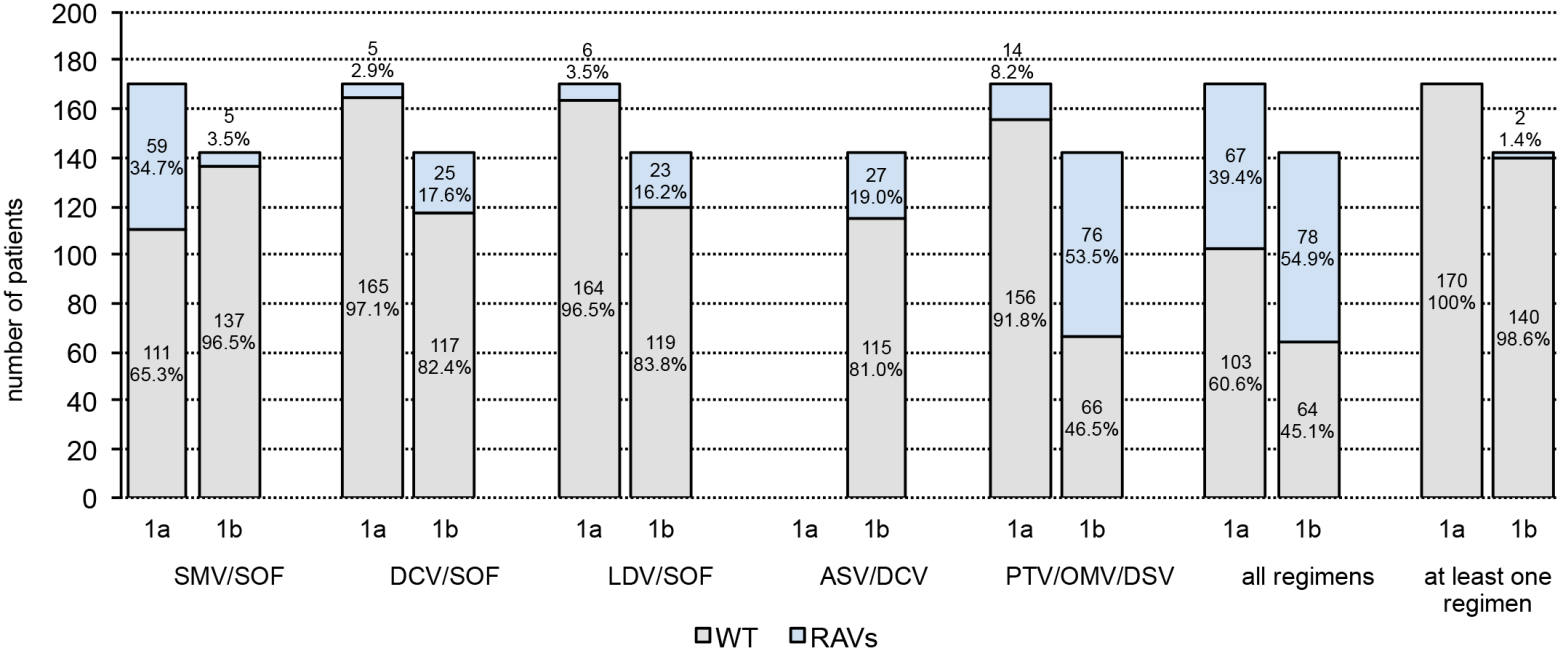
Rate of patients with baseline RAVs for different DAA combination therapies.

Next, we analyzed the proportion of patients who are favourably suited to receive interferon-free treatment regimens under the consideration of baseline RAVs. For the treatment with SMV/SOF, we considered all *NS3* protease mutations as relevant shown in Fig 1. No RAVs (wildtype) were detected in 65.3% of GT1a- and 96.5% of GT1b-infected patients, potentially enabling a SMV/SOF-based therapy (Fig 4).

For daclatasvir and ledipasvir the resistance profiles were similar (see above). The vast majority of GT1a-infected individuals contain no RAVs within *NS5A* supporting a DCV/SOF (97.1%) or LDV/SOF therapy (96.5%). The Y93F variant detected in one GT1a patient is known to confer resistance only towards LDV [22] and L31F in GT1b is low-level resistant towards DCV and OMV but was not described for LDV [18, 20]. Therefore the resistance profiles of these substances are slightly deviating and 82.4% and 83.8% of GT1b-infected patients were eligible to receive DCV/SOF or LDV/SOF respectively (Fig 4).

As the combination therapy consisting of asunaprevir and daclatasvir (ASV/DCV) showed reduced efficacy against GT1a [44], this therapy is approved only for GT1b. 81.0% of GT1b-infected individuals had no *NS3* or *NS5A* RAVs conferring resistance to ASV or DCV (Fig 4). Of the 27 patients with RAVs, three persons carried RAVs within both *NS3* and *NS5A* (not shown) and the majority of RAVs can be attributed to the NS5A Y93H mutation (Fig 2).

For paritaprevir, ombitasvir and dasabuvir triple therapy, combined RAVs within *NS3*, *NS5A and NS5B* were investigated and wildtype variants for all three regions were identified in 91.8% of GT1a and 46.5% of GT1b patients (Fig 4). In GT1a RAVs mainly consisted of mutations within *NS5A* (M28V, Q30H, Y93C) or *NS5B* (C316Y, S556G/N/R). Of note, RAVs within both *NS5A* and *NS5B* were displayed by one GT1a and 10 GT1b-infected patients (data not shown). In GT1b, Y93H within *NS5A* as well as C316N and S556G within *NS5B* were the dominating mutations (Figs 2 and 3). The only *NS3* RAV relevant for this therapy was D168E (n = 2, GT1b) (Fig 1).

Furthermore, our interest was to identify patients without any restrictions to receive the described therapy regimens under the consideration of RAVs. Wildtype variants with respect to all treatment regimens were displayed by 60.6% of patients with GT1a and 45.1% with GT1b (Fig 4). These patients are in principle suited for all therapies. In GT1a, RAVs mainly consisted of the *NS3* Q80K mutation relevant for SMV/ASV (34.7%, Fig 1A) and to a much lesser extend of the OMV-relevant M28V variant within *NS5A* (3.5%, Fig 2A). *NS5B* RAVs were detected rarely in GT1a-infected individuals. Vice versa in GT1b, RAVs within *NS3* occurred relatively infrequently and the *NS5A* Y93H mutation (14.1%, Fig 2B) as well as the *NS5B* C316N/S556G mutations dominated (42.3%, Fig 3B).

Another important point was the evaluation of patients who are not eligible to receive any approved IFN-free treatment regimen when baseline RAVs are considered. Therefore, the proportion of patients who are wildtype for at least one therapy was determined. All GT1a and 98.6% of GT1b-infected patients displayed wildtype variants for at least one therapy option (Fig 4). Only two individuals with GT1b had RAVs with relevance for all substances. Both patients exhibited *NS3* RAVs at position 80 (Q80K, Q80R+D168E) as well as the *NS5A* Y93H mutation which are associated with resistance to all of the above treatment regimens. When the single therapy regimens are compared, DCV- and LDV-based therapies are the treatment form for which the most patients have favourable preconditions with respect to the complete absence of baseline RAVs.

## Discussion

Before antiviral treatment, *NS3*, *NS5A* and non-nucleoside polymerase inhibitor *NS5B* RAVs pre-exist at different frequencies in DAA-naïve patients and these variants may be selected rapidly during treatment with DAAs with the possible consequence of a viral breakthrough and treatment failure [27, 42, 45]. Therefore, resistance testing at baseline may be useful to help to identify the DAA which is the best treatment option for each patient, as all-oral DAA therapies are associated with high costs [45]. However, which frequencies of pre-existing RAVs within the HCV quasispecies and which level of resistance of pre-existing RAVs may contribute to treatment failure is not completely clarified [42, 45]. Recently, baseline *NS5A* RAVs were investigated by deep sequencing using different cut off levels (1% to 20%) and the SVR12 rates after LDV/SOF treatment were similar independent of the frequency of RAVs. However, a reduction of the SVR12 rate was observed in treatment-experienced patients treated for 12 weeks only who had high-level resistant *NS5A* RAVs at baseline. In a subsequent study it turned out that also additional negative treatment response predictors may be of importance as here only in treatment-experienced HCV genotype 1a patients with cirrhosis and high level resistant baseline RAVs virologic response to sofosbuvir plus ledipasvir was significantly reduced [46]. For the combination of sofosbuvir with daclatasvir insufficient resistance data are available but it seems that also here in patients with cirrhosis SVR rates are reduced in patients with baseline *NS5A* RAVs [47]. Finally, for the combination of sofosbuvir with simeprevir the importance Q80K as the major baseline RAV became visible only in the presence of additional factors like shortening treatment duration to 8 weeks or the presence of cirrhosis [48].

In the present study, we applied population-based sequencing to evaluate the prevalence of *NS3*, *NS5A* and *NS5B* RAVs in a cohort of 312 DAA-naïve Caucasian patients in order to identify the eligibility to different treatment regimens under the solely consideration of RAVs.

Within *NS3*, the low-level resistant Q80K mutation, which is based on available data only relevant for SMV and ASV, was most prevalent (34.7% GT1a, 2.1% GT1b) and this is in line with another recent study of European patients which also showed geographical variations of the Q80K prevalence [49]. Patients with the Q80K polymorphism, who were treated with simeprevir in combination with PEG-IFN-α/ribavirin achieved lower SVR rates in comparison to those without Q80K [31] and Q80K was detected in single patients in association with viral breakthrough during ASV/DCV therapy [50]. Moreover, Q80K was also associated with reduced SVR rates in patients treated with sofosbuvir and simeprevir (see above) [51]. Other RAVs (Q80R/D168E) were found in single patients in accordance with other reports [27, 31, 41].

Regarding *NS5A* RAVs, Y93H in GT1b was the primary variant identified (14.1%) which confers resistance to DCV, LDV and OMV. Other researchers also determined Y93H as most frequent baseline *NS5A* RAV in GT1b (6–23%), followed by L31M (3–4%) [22, 52, 53], whereas *NS5A* RAVs occurred at low frequencies in GT1a [27], as shown in our study. Furthermore, the overall number of GT1 *NS5A* RAVs (11.9%) was similar compared to prevalences (11–18%) described using population-based and deep sequencing approaches [5–7]. Interestingly, the majority of GT1a *NS5A* RAVs was intermediate to high-level resistant to DCV, LDV and OMV (M28V, L31M and Y93C/F/N, low-level resistant was only Q30H to OMV). Whereas in GT1b more low- to intermediate resistant variants (Y93H was the only intermediate to high level-resistant variant) were detected. Differences in the level of resistance depending on the HCV subtype were described for Y93H and L31M which are low- to intermediate-level resistant to the majority of NS5A inhibitors in GT1b but confer high level resistance in a GT1a backbone [19, 21, 22, 36]. However, while we detected no Y93H variants in GT1a-infected patients, pre-existing RAVs at position Y93 in GT1a isolates encoded for C, F or N and are associated with high level of resistance.

*NS5B* RAVs with relevance for the non-nucleoside inhibitor dasabuvir were detected infrequently in GT1a. Whereas in GT1b, *NS5B* RAVs were found in nearly one half of individuals (C316N, S556G and both together) conferring low to medium resistance. In mixed cohorts consisting of American and European patients RAVs occurred at frequencies of 11–18% (C316N) and 0.5–16% (S556G) at baseline in GT1b samples [27, 33]. Interestingly, we found in Caucasian patients a much higher prevalence of C316N (35.2%).

We also correlated single RAVs at each position in *NS3*, *NS5A* and *NS5B* with the presence of cirrhosis and the stage of fibrosis. Interestingly RAVs at position L31 in *NS5A* correlated significantly (*P*<0.05) with cirrhosis in GT1b-infected patients (data not shown). As the number of patients with cirrhosis is relatively small in the present study this result should be confirmed in future investigations. Furthermore, it would be interesting to see whether also the selection of L31 variants in patients with treatment failure to DAA regimens is associated with the presence of cirrhosis.

In the present study, all RAVs detectable by population-based sequencing were investigated. As part of these also the prevalence of minor RAVs within *NS3*, *NS5A* and *NS5B* visible by double peaks in the electropherograms was investigated. Altogether, these minor variants were rare and were found in 15 cases only (n = 1, *NS3*; n = 12 *NS5A*; n = 2 *NS5B*). The most common polymorphic site was position Y93 within the *NS5A* gene (see S1 Table).

High SVR rates of more than 90% were achieved in patients treated with various DAA combination regimens [5–7, 47, 48, 51, 54]. This may lead to the assumption that baseline resistance testing might not be necessary. However, for many DAA regimens no complete resistance analysis is available [9–12, 47, 54]. In addition, for a number of currently used DAA combinations the importance of baseline RAVs for virologic treatment response mainly together with the presence of other negative treatment predictors was shown (see above) [8, 22, 46, 48, 51, 55].

Therefore, we analyzed the proportion of patients who are suited to receive a certain available treatment regimen under the primary consideration of baseline RAVs. Although the prevalence of *NS3*, *NS5A* and *NS5B* RAVs was investigated in a large number of studies from mainly American patients [27], no conclusions concerning the applicability of interferon-free DAA combination therapies were drawn in these studies. In the present study, the proportion of patients was determined, who do not have any RAVs conferring resistance to the described substances. Interestingly, nearly two thirds of GT1a and one half of GT1b patients were wildtype for all HCV regions which would enable any regimen. The decision for a respective therapy could be made on the basis of other factors like the tolerability of side effects and the overall costs. The contrary situation, included the determination of the percentage of patients who have unfavourable preconditions to receive any of the above DAA-based therapies based on baseline RAVs. Importantly, for all patients with GT1a and for 98.6% of GT1b-infected individuals, the investigation of baseline RAVs leads to the identification of at least one putative treatment regimen without any baseline RAV to an available DAA combination.

In this study we did not investigate long PCR products including the *NS3* to *NS5B* genes in one fragment. Due to the different lengths of the single *NS3*, *NS5A* and *NS5B* PCR products, the amplification efficiency may have not been totally equal which could in principle lead to a slightly deviating estimation of *NS3*, *NS5A* and *NS5B* covariants. It may appear contradictory that the number of RAVs was lower in GT1a-infected patients compared to GT1b in our study as higher SVR rates were observed in patients with GT1b compared to GT1a for many DAA combinations [22, 56]. We think that these differences could be attributed to the fact that the majority of RAVs detected in GT1b are low-level resistant variants in *NS5B* (C316N and S556G). These variants probably have a weak influence on treatment efficacy, as shown for low-level resistant *NS5A* RAVs [22]. Whereas in GT1a, although the overall number of RAVs was lower, RAVs (except Q80K) were intermediate to high-level resistant which might explain the slightly reduced treatment efficacy of DAAs in GT1a. Furthermore, many DAAs were developed and optimized based on a GT1b cell culture system.

Under consideration of exclusively the presence of baseline RAVs, for treatment of GT1b SMV-based therapies may be more suited, as RAVs occurred rarely in *NS3* but were detectable relatively frequently in *NS5A* and *NS5B* of GT1b-infected patients. For GT1a-infected patients, the treatment of choice may preferably consist on DCV/LDV in combination with SOF, as RAVs within *NS5A* and *NS5B* emerged infrequently in GT1a. For DAA triple therapy with PTV/OMV/DSV the likeliness of at least one pre-existing RAV is increasing (8.2% for GT1a and 53.5% for GT1b). However, also the overall barrier to resistance with three DAAs is increasing. Thus, the importance of single RAVs may be reduced in a three DAA regimen which is supported by the extremely high SVR rates in HCV genotype 1b patients with a three DAA therapy (99%) [12]. Nevertheless, our study is limited due to the fact, that patients were not treated with DAAs. Because of this, no conclusions could be drawn regarding the impact of baseline RAVs on the SVR rate with and without additional negative treatment predictors and further prospective studies are needed for these investigations.

In conclusion, when only baseline RAVs are taken into account, an appropriate interferon-free treatment option without the presence of RAVs could be applied to nearly all patients. Therefore, baseline resistance testing might be considered together with available DAA regimens and other predictors of response to identify an effective and individual treatment option for patients.

## Supporting Information

S1 Table Minority RAVs detected in *NS3*, *NS5A* and *NS5B*. (DOCX)
