# Supplementary material for: Consideration of Viral Resistance for Optimization of Direct Antiviral Therapy of Hepatitis C Virus Genotype 1-Infected Patients
Source: PLoS One. 2015 Aug 28;10(8):e0134395. doi: 10.1371/journal.pone.0134395 (PMC4552686; doi:10.1371/journal.pone.0134395)
Supplement: S1 Table — (DOCX) [file pone.0134395.s001.docx]

S1 Table: Minority RAVs detected in *NS3*, *NS5A* and *NS5B*.

| Patient | HCV-  GT | Region | Variant | Estimated frequency from electropherogram | Overall number of patients with respective variant |
| --- | --- | --- | --- | --- | --- |
| #23 | 1b | NS3 | Q80Q/K | Q: 40%, K:60% | n=3 with Q80K in GT1b |
| #10 | 1a | *NS5A* | M28M/V | M: 40%, V: 60% | n=6 with M28V in GT1a |
| #15 | 1b | *NS5A* | L31L/M | L: 70%, M: 30% | n=3 with L31M in GT1b |
| #23 | 1b | *NS5A* | L31L/F | L: 70%, F: 30% | n=2 with L31F in GT1b |
| #243 | 1a | *NS5A* | Y93Y/F | Y: 50%; F: 50% | n=1 with Y93N in GT1a |
| #248 | 1a | *NS5A* | Y93Y/C | Y: 70%, C: 30% | n=1 with Y93C in GT1a |
| #150 | 1b | *NS5A* | Y93Y/H | Y:60%, H: 40% | n=20 with Y93H in GT1a |
| #154 | 1b | *NS5A* | Y93Y/H | Y: 50%, H: 50% |  |
| #231 | 1b | *NS5A* | Y93Y/H | Y:70%, H: 30% |  |
| #42 | 1b | *NS5A* | Y93Y/H | Y: 60%, H:40% |  |
| #16 | 1b | *NS5A* | Y93Y/H | Y:60%, H: 40% |  |
| #252 | 1b | *NS5A* | Y93Y/H | Y: 50%, H: 50% |  |
| #26 | 1b | *NS5A* | Y93Y/H | Y: 40%, H: 60% |  |
| #252 | 1b | *NS5B* | S556S/G | S: 50%, G: 50% | n=29 with S556G in GT1b |
| #24 | 1b | *NS5B* | S556S/G | S: 30%, G: 70% |  |
